# *Fstl1* is involved in the regulation of radial glial scaffold development

**DOI:** 10.1186/s13041-015-0144-8

**Published:** 2015-09-17

**Authors:** Rui Liu, Yang Yang, Junhui Shen, He Chen, Qianqian Zhang, Ru Ba, Yongjie Wei, Kai-Cheng Li, Xu Zhang, Chunjie Zhao

**Affiliations:** Key Laboratory of Developmental Genes and Human Diseases, MOE, Department of Anatomy and Neuroscience, Medical School, Southeast University, Nanjing, 210009 China; Institute of Neuroscience and State Key Laboratory of Neuroscience, Shanghai Institutes for Biological Sciences, Chinese Academy of Sciences, Shanghai, 200031 China; Center of Depression, Beijing Institute for Brain Disorders, Beijing, 100069 China

## Abstract

**Background:**

Radial glial cells (RGCs), the instructive scaffolds for neuronal migration, are well characterized by their unique morphology and polarization; these cells extend elongated basal processes to the pial basement membrane (BM) and parallel to one another. However, little is known about the mechanisms that underlie the developmental regulation and maintenance of this unique morphology.

**Results:**

Here, by crossing *Fstl1*^*fl/fl*^ mice with an *EIIa-Cre* line, we identified a new role for the secreted glycoprotein Follistatin like-1 (FSTL1). The ablation of *Fstl1* in both of its cortical expression domains, the ventricular zone (VZ) and the pia mater, resulted in RGC morphologic disruption; basal processes were not parallel to each other, and endfeet exhibited greater density and branching. However, *Fstl1* deletion in only the VZ in the *Emx1*^*IREScre*^; *Fstl1*^*fl/fl*^ line did not affect RGC morphology, indicating that FSTL1 derived from the pia mater might be more important for RGC morphology. In addition, upper-layer projection neurons, not deeper-layer projection neurons, failed to reach their appropriate positions. We also found that BMP, AKT/PKB, Cdc42, GSK3β, integrin and reelin signals, which have previously been reported to regulate RGC development, were unchanged, indicating that *Fstl1* may function through a unique mechanism.

**Conclusions:**

In the present study, we identified a new role for FSTL1 in the development of radial glial scaffolds and the neuronal migration of upper-layer projection neurons. Our findings will improve understanding of the regulation of RGC development and neuronal migration.

**Electronic supplementary material:**

The online version of this article (doi:10.1186/s13041-015-0144-8) contains supplementary material, which is available to authorized users.

## Background

Radial glial cells (RGCs), which serve as progenitor cells and as the scaffolds for neuronal migration, are important for neocortical morphogenesis [1, 2]. During cortical development, the soma of an RGC lies in the ventricular zone (VZ), with a specialized apical domain in contact with the ventricular surface, while an elongated basal process extends from its cell body through the entire cortical wall. The tips of its basal processes, which are called the basal endfeet, attach to the pial basement membrane (BM) [1, 3]. Despite the diverse functions of RGCs, the mechanisms underlying their development remain unclear. In the VZ, apical signalling molecules, such as beta-catenin, ZO-1, and Cdc42, are reportedly crucial for the integrity of intercellular adhesion, the specialization of the apical membrane domain and the division pattern of RGCs [4, 5]. The development of polarity and the correct orientation of RGCs’ basal processes depend on the activity of adenomatous polyposis coli (APC) and GSK3 [5–7]. Previous studies have provided evidence that the basal radial processes of RGCs receive signals from the meninges and adjacent Cajal-Retzius (CR) cells [8, 9]. However, the mechanisms that regulate the development of RGC processes remain unclear.

FSTL1 has been reported to be involved in the fate determination and maturation of epithelial cells [10, 11]. However, the function of *Fstl1* during cortical development remains unknown. Previously, we have observed that *Fstl1* is enriched in the VZ and the pia mater of the developing cerebral cortex [12]. Considering the unique morphology of RGCs and the distinct expression pattern of FSTL1, it is quite plausible that *Fstl1* is involved in the development of RGCs. In the present study, we report that ablation of *Fstl1* in both the VZ and the pia mater led to abnormal radial scaffold morphology and further led to the abnormal distribution of cortical pyramidal neurons.

## Results

### Disruption of *Fstl1* results in an abnormal distribution of cortical upper-layer neurons

**Fig. 1:**
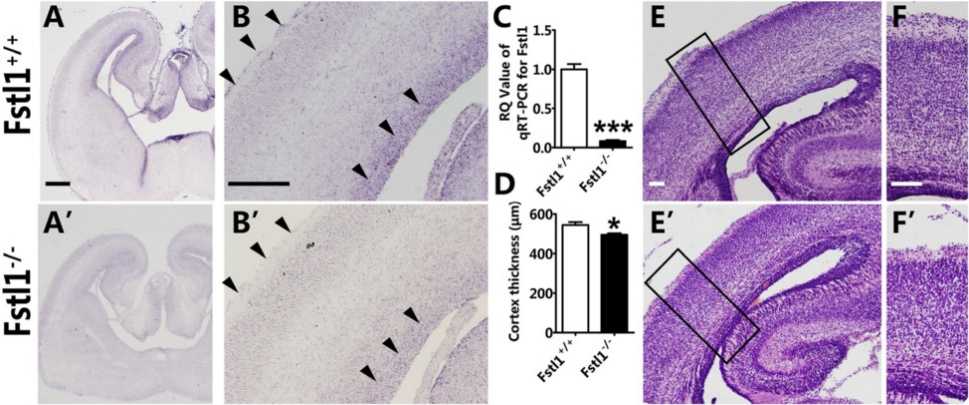
Disruption of *Fstl1* resulted in morphological alteration of the developing cortex. **a** to **b**’ The significant reduction of the *Fstl1* mRNA level in the *Fstl1* ^−/−^ cerebral cortex. At E16.5, strong *Fstl1* mRNA signal can be detected in the VZ and pia mater in the WT cerebral cortex (**a** and **b**), but *Fstl1* mRNA cannot be detected in the *Fstl1* ^−/−^ cerebral cortex (**a**’ and **b**’). **c** qRT-PCR for *Fstl1* from E14.5 dorsal cerebral cortical extracts confirms the dramatic reduction of *Fstl1* mRNA levels (***p < 0.001, *n* = 5). **d** The thickness of the cortical wall is reduced in the *Fstl1* ^−/−^ cerebral cortex. The data are the mean ± s.e.m.: 544.3 ± 14.7 μm for WT, *n* = 6; and 494.8 ± 7.1 μm for *Fstl1* ^*−/−*^ *, n* = 5, *p < 0.05. **e** to **f**’ HE staining shows the defective arrangement of upper-layer neurons in *Fstl1* ^*−/−*^ cortices at E18.5 (**e**’ and **f**’). Scale bars: 100 μm

To better understand the development of radial glial scaffolds and to address the function of *Fstl1* during cortical morphogenesis, *Fstl1* was ablated by crossing the *EIIa-Cre* line with *Fstl1*^*fl/fl*^. In situ hybridization showed a dramatic reduction of *Fstl1* mRNA in the VZ and the pia mater in *Fstl1*^−/−^ brains at E16.5 (Fig. 1a–b’). The disruption efficiency was also confirmed by quantitative real-time polymerase chain reaction (qRT-PCR) (Fig. 1c). Because the homozygous null pups died within a few hours of birth, the observations were conducted at embryonic stages. HE staining at E18.5 showed a slight reduction in the thickness of the cortical wall in the *Fstl1*^*−/−*^ embryos compared with that of the WT cortical walls, but the overall architecture of the brain did not appear to be severely affected (Fig. 1d–f’). The cortical neurons, especially the upper-layer neurons, were not tightly arranged, but the overall lamination did not appear to be obviously disturbed (Fig. 1e–f’). These findings suggest that the loss of *Fstl1* affects the accurate localization of cortical neurons.

**Fig. 2:**
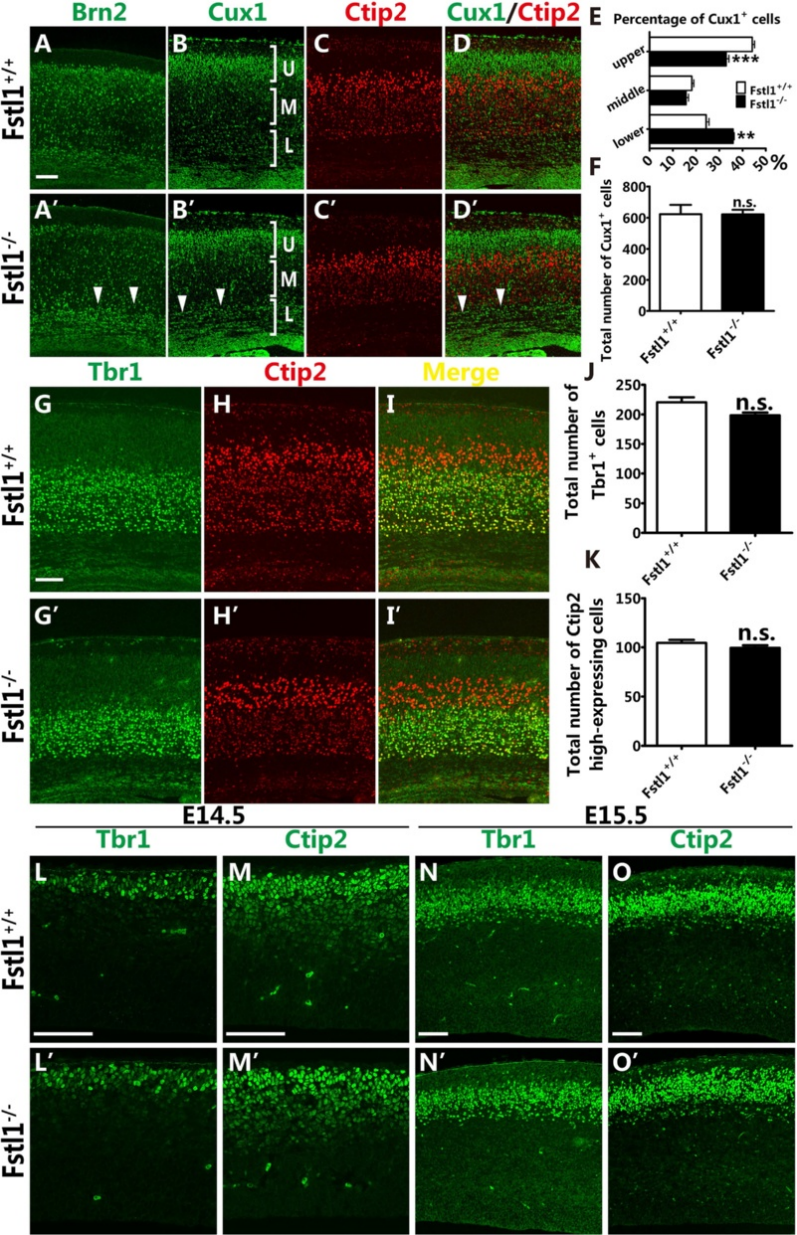
Disruption of *Fstl1* results in an abnormal distribution of upper-layer but not deeper-layer cortical neurons. **a** to **d**’ The abnormal distribution of upper-layer neurons in the *Fstl1* ^−/−^ cerebral cortex. Immunostaining for Brn2 (*arrowheads*, [**a**’]) and Cux1 (*arrowhead*, [**b**’]) at E18.5 shows many upper-layer neurons still residing in the IZ and subplate after *Fstl1* ablation (*arrowhead* in **a**’ and **b**’). Co-immunostaining for Cux1 (**b**, **b**’, **d** and **d**’) and Ctip2 (**c**, **c**’, **d** and **d**’) at E18.5 shows the abnormal distribution of upper-layer neurons in the deeper area. **e** The distribution of Cux1^+^ neurons at E18.5. More Cux1^+^ neurons accumulated in the lower area at E18.5 (24.31 ± 1.26 % for WT, *n* = 4; 35.94 ± 0.48 % for *Fstl1* ^*−/−*^, *n* = 4, **p < 0.01), while fewer Cux1^+^ neurons were present in the upper area (44.14 ± 1.03 % for WT, *n* = 4; 32.98 ± 1.16 % for *Fstl1* ^*−/−*^, *n* = 4, ***p < 0.001). **f** The total number of Cux1^+^ neurons per area at E18.5. The data are the mean ± s.e.m.: 623.5 ± 58.5 for WT, *n* = 4; and 622.3 ± 29.1 for *Fstl1* ^*−/−*^, *n* = 4, *p* = 0.99. **g** to **i**’ Double immunostaining for Tbr1 and Ctip2 at E18.5 showed that the distributions of layer VI and layer Va neurons are unchanged in the *Fstl1* ^*−/−*^ cortex compared to the WT cortex. **j** and **k** The total numbers of Tbr1^+^ layer VI neurons (**j**) and layer Va neurons with high Ctip2 expression per area at E18.5 (**k**). The Tbr1^+^ neuron data are presented as the mean ± s.e.m.: 220.5 ± 8.4 for WT, *n* = 4; and 198.4 ± 4.7 for *Fstl1* ^*−/−*^, *n* = 4, *p* = 0.07. The high Ctip2 expression neuron data are presented as the mean ± s.e.m.: 104.5 ± 3.0 for WT, *n* = 5; and 99.4 ± 2.6 for *Fstl1* ^*−/−*^, *n* = 5, *p* = 0.24. **l** to **o**’ The comparable distribution of deeper-layer neurons at early embryonic stages in WT and *Fstl1* ^*−/−*^ mice. Immunostaining for the deeper-layer neuronal markers Tbr1 (**l**, **l**’, **n** and **n**’) and Ctip2 (**m**, **m**’, **o** and **o**’) at E14.5 (**l** to **m**’) and E15.5 (**n** to **o**’) shows that the migration and distribution of deeper-layer neurons are unchanged in the *Fstl1* ^*−/−*^ cortex compared to the WT cortex. Scale bars: 100 μm

To assess which groups of neurons were affected during cortical development, the layer-specific markers Brn2 (II-III), Cux1 (layers II-IV), Ctip2 (V-VI), and Tbr1 (VI) were used. At E18.5, many Cux1^+^ and Brn2^+^ cells still resided in the intermediate zone (IZ) and subplate in the *Fstl1*^*−/−*^ mice (Fig. 2a’ and b’), but in the WT mice, the majority of the Cux1^+^ and Brn2^+^ cells had arrived at the upper cortical plate (Fig. 2a and b). Double immunostaining for Cux1 and Ctip2 also showed that at E18.5, a number of Cux1^+^ neurons remained in deeper cortical areas in the *Fstl1*^*−/−*^ mice compared with the WT mice (Fig. 2c–d’). The statistical analysis showed that at E18.5, only 32 % of the Cux1^+^ neurons had arrived in the upper area in the *Fstl1*^*−/−*^ cortex compared with 44 % in the WT cortex; in addition, 35 % of the Cux1^+^ neurons were accumulated in the lower area in the *Fstl1*^*−/−*^ cortex but only 24 % were in the WT cortex (Fig. 2e). However, the total number of Cux1^+^ neurons did not differ significantly between the *Fstl1*^*−/−*^ and WT mice (Fig. 2f). Our data showed that the migration of upper-layer projection neurons was disrupted after *Fstl1* deletion. In the deeper-layer projection neurons, the distributions of Tbr1^+^ layer VI neurons and Ctip2-high expression layer Va neurons in the *Fstl1*^*−/−*^ mice were comparable with those in the WT mice (Fig. 2g–i’), and the total numbers of Tbr1^+^ neurons and Ctip2 high-expression neurons did not differ (Fig. 2j and k). The lamination of the deeper layers seemed similar between the *Fstl1*^*−/−*^ and WT mice, and there was a clear boundary between layer VI and layer Va (Fig. 2g–i’).

To examine whether the radial migration of deeper-layer neurons was delayed at earlier embryonic stage and finally recovered at E18.5, cortices were harvested at E14.5 and E15.5 and immunostained with the deeper-layer-specific markers Tbr1 and Ctip2. Our data revealed that Tbr1^+^ and Ctip2^+^ neurons in the *Fstl1*^*−/−*^ cortex were positioned appropriately in the emerging cortical plate at E14.5 and in the deeper part of the cortical plate at E15.5, comparable with the positions observed in the WT cortex, as shown in Fig. 2l–o’. Thus, the migration of deeper-layer projection neurons was not obviously disrupted. Taken together, our data showed that the migration of upper-layer projection neurons was more severely impaired at perinatal stages following *Fstl1* ablation.

To test whether the ablation of *Fstl1* resulted in cortical interneuron defects, we examined the distribution of interneurons derived from the medial ganglionic eminence (MGE) and caudal ganglionic eminence (CGE). In situ hybridization for the neuropeptide somatostatin (*SST*) and the LIM homeobox protein 6 (*Lhx6*) showed that the distribution of MGE-derived interneurons was unchanged in *Fstl1*^*−/−*^ mice compared to WT mice. Immunostaining for COUP-TFII also indicated that the overall migration and lamination of CGE-derived interneurons were similar to those of the WT interneurons at E18.5 (data not shown). Our data demonstrated that disruption of *Fstl1* had no obvious effects on cortical interneuron development.

**Fig. 3:**
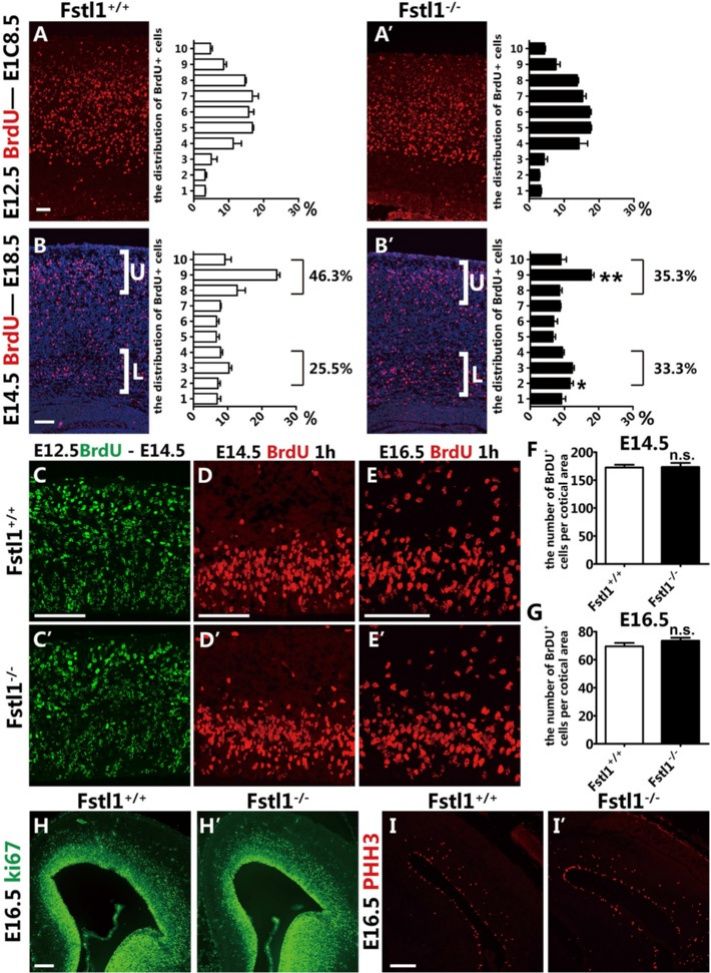
Loss of *Fstl1* results in aberrant positioning of upper-layer cortical neurons. **a** to **b**’ Although similar distributions of E12.5-born cells are observed in the WT (**a**) and *Fstl1* ^*−/−*^ (**a**’) cortices, a greater fraction of cell born at E14.5 (**b**’) are found in deeper cortical layers in the *Fstl1* ^*−/−*^ cortices compared to the WT cortices (**b**). The percentages of cells in the upper layers (bins 8–10) and IZ (bins 2–4) are indicated in the histograms. *n* = 3 per genotype per experimental condition. *p < 0.05, ** *p* < 0.01. The error bars represent the s.e.m. **c** and **c**’ The migration of early-born deeper-layer neurons is unchanged in the *Fstl1* ^*−/−*^ cortex (**c**) compared to that in the WT cortex (**d**). BrdU was injected at E12.5, and the brains were then harvested at E14.5. **d** to **e**’ The number of BrdU^+^ cells was similar in the WT (**d** and **e**) and *Fstl1* ^*−/−*^ cortices (**d**’ and **e**’) at E14.5 (**d** and **d**’) and E16.5 (**e** and **e**’). **f** and **g** The number of BrdU^+^ progenitor cells per entire cortical section at E14.5 (**f**) and E16.5 (**g**). The data are the mean ± s.e.m. At E14.5, 172.4 ± 5.1 for WT, *n* = 5; and 173.5 ± 7.2 for *Fstl1* ^*−/−*^, *n* = 5, *p* = 0.91. At E16.5, 69.5 ± 2.4 for WT, *n* = 3; and 73.5 ± 2.1 for *Fstl1* ^*−/−*^, *n* = 3, *p* = 0.28. **h** to **i**’ Immunolabelling with anti-Ki67 (**h** and **h**’) and anti-pHH3 (**i** and **i**’) antibodies confirmed that the proliferation of progenitor cells was not obviously changed at E16.5. Scale bars: 100 μm

To further elucidate the role of *Fstl1* in neuronal migration, 5-bromo-2-deoxyuridine (BrdU) birth-dating was employed. BrdU was injected at E12.5 to label the deeper-layer neurons or at E14.5 to label the upper-layer neurons. The brains were then harvested at E18.5. In the mutants, the distribution of deeper-layer BrdU^+^ neurons born at E12.5 was similar to that in the WTs (Fig. 3a and a’). In the WT mice, 46.3 ± 2.3 % of the BrdU^+^ cells that were labelled at E14.5 populated the upper part of the cortex; however, this percentage was reduced to 35.3 ± 3.0 % in the *Fstl1*^*−/−*^ mice. The BrdU^+^ cells were distributed more evenly throughout the cortex, and a population of BrdU^+^ cells remained in the deeper cortical layers and IZ in the *Fstl1*^*−/−*^ mice. In the WT mice, approximately 25.5 ± 1.6 % of the BrdU^+^ cells were distributed in the deeper layers, but this percentage increased to 33.3 ± 1.3 % in the *Fstl1*^*−/−*^ mice (Fig. 3b and b’). To further assess the migration of deeper-layer neurons at earlier developmental stages, BrdU was injected at E12.5, and the brains were then harvested at E14.5. In the mutants, the distribution of BrdU^+^ neurons born at E12.5 was similar to that in the WTs (Fig. 3c and c’). These results indicate that *Fstl1* dysfunction resulted in mis-positioning of the upper- but not deeper-layer projection neurons.

To examine whether *Fstl1* affects neuronal proliferation, acute BrdU labelling was performed at E14.5 and E16.5 (Fig. 3d–e’). The numbers of BrdU-labelled cells did not differ between the *Fstl1*^*−/−*^ pups and their WT littermates (E14.5, 172.4 ± 5.1 for WT, *n* = 5 vs 173.5 ± 7.2 for *Fstl1*^*−/−*^, *n* = 5; E16.5, 69.5 ± 2.4 for WT, *n* = 3 vs 73.5 ± 2.1 for *Fstl1*^*−/−*^, *n* = 3) (Fig. 3f and g), and this result was further confirmed by immunostaining for the pan-proliferation marker Ki67 and the mitotic phase marker phospho-histone H3 (pHH3) (Fig. 3h-i’), which suggests that the deletion of *Fstl1* does not affect the proliferation of progenitor cells. Because upper-layer cortical neurons are primarily derived from intermediate progenitor cells (IPCs) rather than directly from RGCs, we next examined the number of IPCs by immunostaining for Tbr2 [13] and found no obvious differences (Additional file 1: Figure S1), which indicates that the neurogenesis of the upper-layer neurons was also not affected. Additionally, immunostaining for activated Caspase 3 was performed at E14.5, and an obvious excess of cell death was not detected (data not shown).

### The development of radial glial scaffolds is disrupted in *Fstl1*^*−/−*^ cerebral cortices

**Fig. 4:**
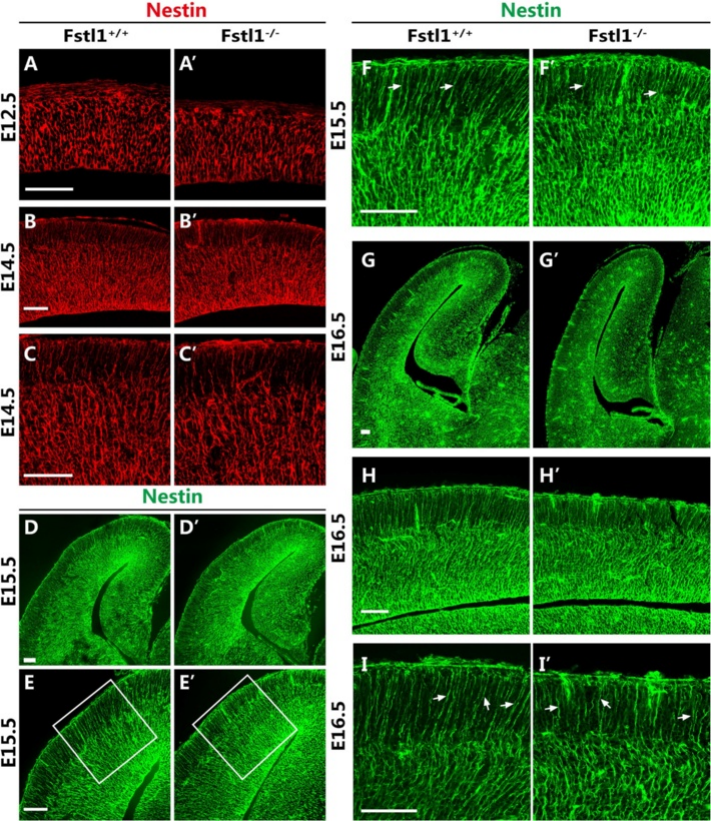
*Fstl1* deletion impairs the morphology of radial glial processes. **a** to **i**’ Nestin immunostaining at E12.5 (**a** and **a**’), E14.5 (**b** to **c**’), E15.5 (**d** to **f**’) and E16.5 (**g** to **i**’). At E12.5, the development of radial glial fibres was comparable between the WT (**a**) and *Fstl1* ^*−/−*^ (**a**’) cortices. **b** to **c**’ At E14.5, many long, parallelly distributed processes spanning the entire cerebral cortex were observed in the WT brains (**b** and **c**). In the *Fstl1* ^*−/−*^ brains, many radial processes were not parallel to one another (**b**’ and **c**’). **d** to **i**’ At E15.5 and E16.5, the same phenomenon was detected in the RGCs. Scale bars: 100 μm

**Fig. 5:**
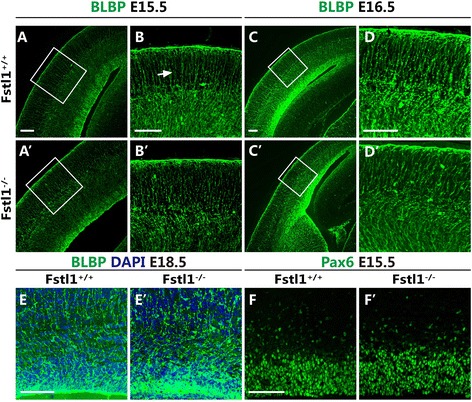
*Fstl1* deletion impairs the morphology of RGC processes but does not disturb the progenitor pool. **a** to **e**’ At E15.5 (**a** to **b**’), E16.5 (**c** to **d**’) and E18.5 (**e** and **e**’), a similar phenomenon in RGC structure was confirmed by immunostaining for BLBP. **f** and **f**’ Pax6 immunostaining showed that the radial progenitor pool was not obviously affected at E15.5. Scale bars: 100 μm

Because the upper-layer neurons reach their final destinations by migrating along radial glial scaffolds [14], we next investigated whether the positional defect of the upper-layer neurons in the *Fstl1*^*−/−*^ mice was due to an abnormality in the RGCs. Immunostaining for nestin and BLBP, which are standard markers for RGCs, was performed. At E12.5, the nestin-stained radial glial scaffolds in the *Fstl1*^*−/−*^ mice appeared to be similar to those in the WT mice, with many radially distributed processes extending throughout the cortical wall (Fig. 4a and a’). However, although many long, radially distributed processes spanned the entire cerebral cortex in the WT mice at E14.5, numerous short radial processes failed to extend perpendicularly through the entire cerebral cortex towards the pial BM in the *Fstl1*^*−/−*^ mice, and the radial scaffolds were not fasciculated as tightly as in the WT mice (Fig. 4b–c’). Similar defects in the radial glial scaffolds were detected at E15.5 and E16.5 (Fig. 4d–i’). Immunostaining for BLBP further confirmed the defects at E15.5 and E16.5 (Fig. 5a–d’). At E18.5, the defects in the RGC processes were still apparent (Fig. 5e and e’). To examine whether the radial glial progenitor pool was affected, immunostaining for the transcription factor Pax6, which labels RGCs [13, 15], was performed, and the progenitor pool was found to be relatively normal (Fig. 5f and f').

**Fig. 6:**
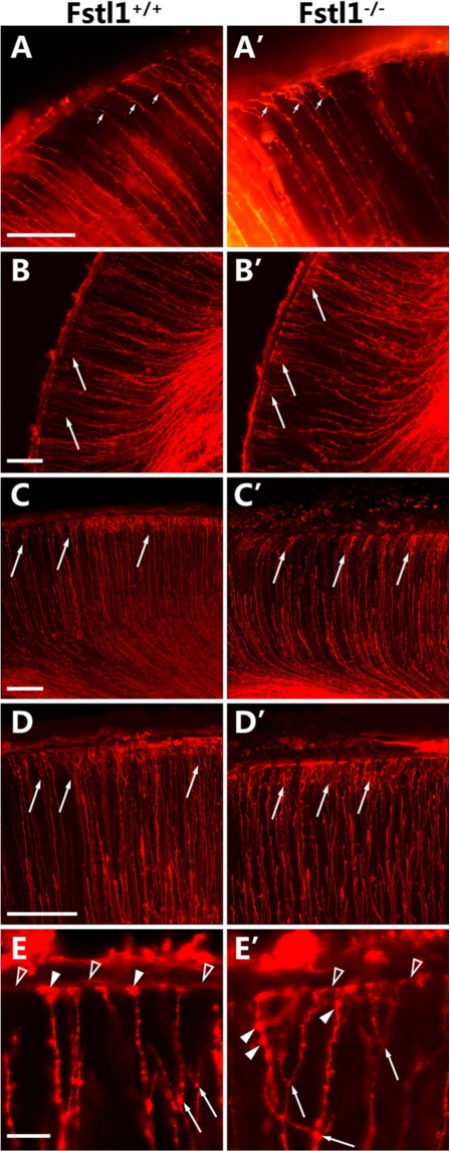
Dysmorphic RGCs with defective branches near the pial BM in the Fstl1-/- mice. RGC scaffolds are shown by DiI labelling at E15.5. (**a** and **a**’) Images were captured with an Olympus BX61 microscope and a DP71 digital camera. (**b** and **b**’) Images were captured with an FV1000 Olympus confocal microscope. (**c** to **d**’) The images are a projection of an 80-μm z-stack collected on an Olympus FV1000 confocal microscope. In the WT cortices, the RGC processes were aligned in parallel and were less branched (**a** and **c**). In the WT cortices, the branches of RGC processes were near the pial BM (**d**). In the Fstl1-/- cortices, many RGC processes were not parallel to one another (**b**’-**d**’), and the branching of their endfeet was more intricate (arrows in **a**’-**d**’). (**e** and **e**’) The images were high magnification of RGC scaffold endfeet. In the WT cortices, the endfeet of RGC processes (arrowhead in **e**) attached to the pial BM (open arrowhead in **e**). In the Fstl1-/- cortices, many RGC scaffold endfeet (arrowhead in **e**’) detached from the pial BM (open arrowhead in **e**’), and the branching of RGC processes was more intricate (arrows in **e**’). Scale bars: 100 μm for **a**-**d**’; 20 μm for **e** and **e**’

To further study the dysmorphic radial glia in the absence of *Fstl1*, the radial glial scaffolds were labelled with DiI at E15.5 and observed with a standard fluorescence microscope (Fig. 6a and a’) or a confocal microscope (Fig. 6b–d’). In the WT brains, the RGC processes were oriented parallel to one another and orthogonal to the pial BM (Fig. 6b and c), and their basal endfeet were less branched (Fig. 6a–d). However, in the *Fstl1*^−/−^ cortices, many RGC processes were not parallel to one another (Fig. 6a’-d’). Furthermore, the branches of the basal endfeet were more intricate and were located farther away from the pial surface than those in the WT cortices (Fig. 6c–d’). Thus, the disruption of *Fstl1* results in abnormal RGC development.

### Basal rather than apical polarity of RGCs is altered in the absence of *Fstl1*

**Fig. 7:**
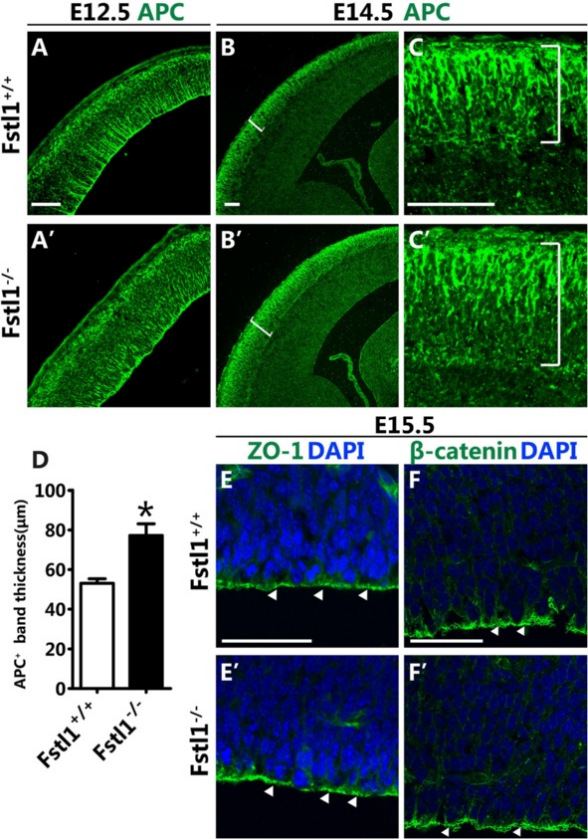
The basal rather than the apical polarity of RGCs is altered in *Fstl1* ^*−/−*^ mice*.* **a** and **a**’ At E12.5, APC is highly expressed at the pially directed tips of the RGCs and at the apical BM of the VZ. The expression pattern of APC is comparable between the WT (**a**) and *Fstl1* ^*−/−*^ (**a**’) brains. **b** to **c**’ At E14.5, a broader APC-positive band was observed in *Fstl1* ^*−/−*^ (**b**’ and **c**’) compared to that of the WT (**b** and **c**). **c** and **c**’ Higher-power views of the boxed areas in **b** and **b**’. **d** The thickness of the APC^+^ band was reduced in the *Fstl1* ^−/−^. Data are presented as the mean ± s.e.m. for the WT (53.06 ± 2.62 μm, *n* = 3) and *Fstl1* ^*−/−*^ (77.20 ± 5.87 μm, *n* = 3) cortices. *p < 0.05. **e** to **g**’ Immunolabelling with anti-ZO-1 (**e** and **e**’) and anti-β-catenin (**f** and **f**’) at E15.5 showed that the apical polarity of the RGCs was not obviously altered. Scale bars: 100 μm for a-c’, f and f’; 50 μm for **e** and **e**’

A defining feature of RGCs is their apical-basal polarity. Recent studies have indicated that disruption of the polarity of RGCs disturbs both the morphology of radial glial processes and neuronal migration [5]. The tumour suppressor APC is highly concentrated at the tips of the processes and in the somas of RGCs and is required for the maintenance and extension of the radial glial processes [7]. To test whether the polarity of RGCs was altered after *Fstl1* ablation, immunostaining for APC was conducted. At E12.5, APC was highly expressed at the pially directed tips of the RGCs and on the apical surface of the VZ in both the *Fstl1*^*−/−*^ and WT mice (Fig. 7a and a’). However, at E14.5, a broader APC-enriched band was observed in the *Fstl1*^*−/−*^ subpial region compared to that in the WT region (Fig. 7b–d), which is consistent with the radial glial endfeet defect observed through DiI labelling (Fig. 6a–d’). Upon examination of the integrity of the pial BM, the attachment site for the RGCs’ basal endfeet [16], no abnormalities were detected in the *Fstl1*^*−/−*^ mice, as demonstrated by laminin A immunoreactivity (Additional file 2: Figure S2 and data not shown). These findings demonstrated that the endfeet of the pially directed, polarised radial glial processes could not be established correctly in the absence of *Fstl1*, although the pial BM itself was not affected.

Previous studies have shown that β-catenin, which is localized at the apical adherens junctions, modulates appropriate expansion of the radial progenitor population [4, 17]. ZO-1, which is a tight junction-associated protein, is enriched at the lateral membrane domain of the interphase radial glial apical domain [18]. To examine whether the apical polarity of the RGCs was affected, immunostaining for β-catenin and ZO-1 was performed. Relatively normal β-catenin and ZO-1 expression patterns were observed in the *Fstl1*^*−/−*^ brains (Fig. 7e–f’), which suggests that the apical polarity was not grossly altered. Taken together, the results showed that the ablation of *Fstl1* led to disruption of the basal rather than the apical polarity of RGCs.

### Ablation of *Fstl1* in both the VZ and the pia mater rather than in only the VZ leads to abnormal RGC morphology

**Fig. 8:**
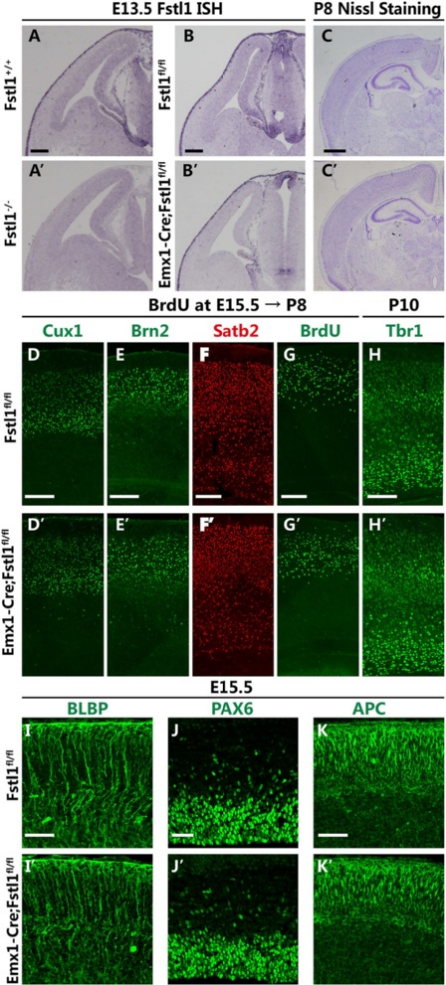
*Fstl1* deletion in the VZ only was not sufficient to induce radial glial dysplasia. **a** to **b**’ A high level of *Fstl1* mRNA can be detected in the pia mater and VZ in the WT (**a**) and *Fstl1* ^*fl/fl*^ (**b**) cortices. In the *Fstl1* ^*−/−*^ mice, *Fstl1* mRNA cannot be detected in the pia mater or the cerebral cortex (**a**’); in the *Emx1* ^*IREScre*^; *Fstl1* ^*fl/fl*^ mice, *Fstl1* expression was absent in the VZ but present in the pia mater (**b**’). **c** and **c**’ Nissl staining showed no remarkable differences in the structure or cellular distribution of the cerebral cortex between the *Fstl1* ^*fl/fl*^ (**c**) and *Emx1* ^*IREScre*^ *; Fstl1* ^*fl/fl*^ (**c**’) mice at P8. **d** to **h**’ Immunolabelling with anti-Cux1 (**d** and **d**’), anti-Brn2 (**e** and **e**’) and anti-Satb2 (**f** and **f**’) antibodies at P8 and with anti-Tbr1 (**h** and **h**’) antibodies at P10 showed that both the upper- (**d** to **f**’) and the deeper-layer (**h** and **h**’) cortical neurons were nicely laminated and eventually migrated to the appropriate cortical layers in the *Emx1* ^*IREScre*^; *Fstl1* ^*fl/fl*^ mice (**d**, **e**, **f** and **h**), similar to observations in the *Fstl1* ^*fl/fl*^ mice (**d**’, **e**’, **f**’ and **h**’). **g** and **g**’ There were no differences in the final destinations of the cells that were labelled with BrdU at E15 between the *Emx1* ^*IREScre*^; *Fstl1* ^*fl/fl*^ and *Fstl1* ^*fl/fl*^ cortices at P8. **i** and **i**’ BLBP immunostaining showed normal radial glial development in the *Fstl1* ^*fl/fl*^ (**i**) and *Emx1* ^*IREScre*^; *Fstl1* ^*fl/fl*^ cortices (**i**’) at E15.5. **j** and **j**’ Pax6 immunostaining showed no obvious differences between the E15.5 *Fstl1* ^*fl/fl*^ and *Emx1* ^*IREScre*^; *Fstl1* ^*fl/fl*^ cerebral cortices. **k** and **k**’ Normal basal branching of the radial glial, as demonstrated by immunolabelling for APC in the *Emx1* ^*IREScre*^; *Fstl1* ^*fl/fl*^ (**k**’) and *Fstl1* ^*fl/fl*^ (**k**) cortices. Scale bars: 100 μm for **a**–**b**’; 200 μm for **c** to **h**’; 50 μm for **i**–**k**’

Because *Fstl1* mRNA is found in both the pia mater and the VZ [12], we sought to determine whether FSTL1 derived from the VZ or the pia mater is more important for the maintenance of polarity in RGCs. *Emx1*^*IREScre*^ mice [19] were employed to disrupt *Fstl1* only in the VZ and not in the pia mater (Fig. 8a–b’). Unexpectedly, no apparent abnormalities were observed in the cortex of *Emx1*^*IREScre*^; *Fstl1*^*fl/fl*^ mice (Fig. 8**c** and **c**’). Both upper-layer and deeper-layer neurons were nicely laminated (Fig. 8d–h’). BrdU birth-dating showed that in the *Fstl1*^*−/−*^ mice, the final distribution of upper-layer neurons born at E15 was comparable with that in the WT mice (Fig. 8g and g’). At E15.5, the radial glial scaffolds, viewed by immunostaining for BLBP, appeared similar to those of the WT mice, with many radially distributed processes that spanned the entire cortical wall and were oriented parallel to one another (Fig. 8i and i’). The Pax6^+^ RGC pool was also unchanged (Fig. 8j and j’). Furthermore, neither the distribution nor the intensity of APC staining was affected (Fig. 8k and k’). These results demonstrated that conditional disruption of *Fstl1* in the VZ was not sufficient to cause the phenotype observed in the *Fstl1*^*−/−*^ brains. Together, our data suggest that FSTL1 derived from the VZ is not necessary for the maintenance of the morphology of RGC processes and that FSTL1 derived from the pia mater is more important.

### The possible mechanisms of *Fstl1* underlying the development of radial glial scaffolds

BMP signalling may be one of the downstream targets of FSTL1 [20, 21], and the deletion of BMP7 results in less radial glia attachment to the pial BM, reduced cortical thickness and impaired neuronal migration [22, 23]. Therefore, we first examined the activity of the canonical BMP signalling pathway by examining the level of phosphorylated SMAD1/5/8. Western blot analysis indicated that the phosphorylation levels in *Fstl1*^*−/−*^ mice were similar to those in WT mice (Additional file 3: Figure S3A). BMP7 qRT-PCR further confirmed that the transcription level of BMP was also unchanged (Additional file 3: Figure S3B). The AKT/PKB signalling pathway is another target of FSTL1. Previous studies have shown that FSTL1 protects cells from apoptosis and induces angiogenesis via phosphorylated AKT [24–26]. However, qRT-PCR and immunoblotting assays showed that the level of AKT was not changed in *Fstl1*^*−/−*^ brains (Additional file 3: Figure S3A and 3C). Furthermore, the activity of the AKT signalling pathway, as monitored by AKT phosphorylation at Ser473, was also unchanged (Additional file 3: Figure S3A). A previous study has shown that Etv5 is a critical target of the FGF/MAPK signalling pathway and has a role in the specification of RGCs [27]. However, neither the expression pattern nor the mRNA level of Etv5 differed between the WT and *Fstl1*^*−/−*^ mice (Additional file 3: Figure S3D, D’ and E). Reelin, which is secreted by CR cells, has been reported to be important for the development of radial scaffolds [28, 29]. However, neither the transcriptional level of *reelin* nor the distribution of Reelin^+^ CR cells was altered in *Fstl1*^*−/−*^ mice (Additional file 3: Figure S3F to G’). Additionally, we examined the mRNA levels of Cdc42 and GSK3β, which have been reported to modulate distinct aspects of radial glial process organization and function, but no differences were detected between the WT and *Fstl1*^*−/−*^ mice (Additional file 3: Figure S3H and I). Integrins are critical for the integrity of the pial BM, to which the radial glial processes attach [30, 31]; however, the mRNA levels of Itgb1, Itga5, and Itga6 did not differ (Additional file 3: Figure S3J–L). We did not observe changes in the expression of genes in the BMP, AKT/PKB, Cdc42, GSK3β, integrin or reelin signalling pathways in the *Fstl1*^*−/−*^ mice, suggesting that FSTL1 may regulate radial glial development through a unique mechanism. Further study is required to elucidate this mechanism.

## Discussion

In the present study, we report that *Fstl1* is involved in the development of radial glial basal processes and in the migration of upper-layer pyramidal neurons in the developing cerebral cortex. Furthermore, the ablation of FSTL1 in both the VZ and the pia mater rather than only in the VZ led to abnormal RGC morphology, indicating that VZ-derived FSTL1 is not necessary for the development of RGC scaffolds and that FSTL1 derived from the pia mater is more important.

### The role of *Fstl1* in the development of radial glial scaffolds

Previous studies have demonstrated that proper apical-basal polarity and tight anchorage to the pial BM are critical for the functions of RGCs [5, 7, 8, 32]. Radial glial development has been reported to be regulated by both intrinsic factors, such as asymmetric protein distribution, and extrinsic factors, such as growth factors and other diffusible ligands [8, 33, 34]. In recent years, several studies have shown that the meninges regulate the survival, proliferation, differentiation and neurogenic properties of RGCs by releasing diffusible factors, such as CXCL12, BMP7 and retinoic acid [9, 23, 35]. Here, we found that *Fstl1* is required for the development of radial glial scaffolds and the basal polarity of RGCs. Interestingly, the disruption of FSTL1 in the VZ did not affect the morphology of radial glial scaffolds, indicating that FSTL1 derived from the pia mater rather than the VZ might play a more critical role during this process and that the role of *Fstl1* in the development of radial glial scaffolds is most likely non-cell-autonomous. Additionally, previous studies have suggested that meningeal cells organize the pial BM, a critical anchor point for radial glial scaffolds. Impaired pial BM integrity leads to RGC scaffold detachment and the apoptotic death of RGCs. However, our data indicates that *Fstl1* is not indispensable for the integrity of the pial BM during the overall embryonic stage. One possible explanation is that FSTL1 produced by the meninges might be critical for the interaction of RGC basal endfeet with the BM, although we cannot exclude the possibility that FSTL1 regulates the organization of the RGC scaffolds directly. Further studies that employ conditional disruption of *Fstl1* in the pial BM will help to elucidate the details of the role of *Fstl1* in the pial BM.

The finding that RGC proliferation was not affected by the ablation of *Fstl1* was also notable. Furthermore, neither the RGC progenitor pool nor the number of IPCs was disturbed, which indicates that the differentiation of RGCs was also unaffected. Our findings demonstrate a new role of *Fstl1* in the maintenance of radial glial scaffolds but not the proliferation and differentiation of RGCs and thus will increase understanding of the molecular mechanisms of RGC development.

### *Fstl1* is required for glial-guided neuronal migration

Our results show that the deletion of *Fstl1* led to mis-positioning of upper-layer projection neurons. Notably, at E18.5, the distributions of deeper-layer projection neurons were not obviously affected. The lamination of layers V and VI seemed normal. One possible explanation is that neuronal migration of all layer neurons, including the upper- and deeper-layer projection neurons, has been delayed. However, the results of our birth-dating and immunostaining of deeper-layer neurons at earlier embryonic stages demonstrated that the migration of deeper-layer neurons was normal after the ablation of *Fstl1*, indicating that Fstl1 is not required for the migration of deeper-layer projection neurons.

Upper-layer neurons are known to preferentially migrate to their final location in a radial-glia-dependent manner, but the deeper-layer neurons likely migrate in a radial-glia-independent manner [36, 37]. Thus, the impaired upper-layer projection neuron migration in the *Fstl1*^*−/−*^ mice was probably the consequence of the abnormalities in the RGCs. It is well known that cortical interneurons migrate tangentially from the ventral telencephalon to the neocortex in a radial-glia-independent manner during embryonic development and then reach their final destination postnatally by radial migration. Here, no distribution defects were observed in our mutants, suggesting that Fstl1 is not important for the development of interneurons. Our data will help to elucidate the mechanisms of upper-layer neuronal migration.

### The possible molecular mechanism of *Fstl1* regulating the development of radial glial scaffolds

Recently, several studies have revealed that BMP signalling and the AKT pathway are the main downstream targets of FSTL1 [20, 21, 26, 38, 39]. However, in the present study, no changes were detected in either pathway after the ablation of *Fstl1*. Reelin is a key regulator of the development of RGCs [29, 40]. However, reelin expression was also unchanged. A likely explanation is that FSTL1 does not function via either the reelin-mediated pathway or the BMP or AKT pathways, and further studies are required to elucidate the molecular mechanism.

## Conclusion

In the present study, we identified a new role for FSTL1 in the development of radial glial scaffolds and glial-guided neuronal migration. Disruption of *Fstl1* resulted in an abnormal distribution of cortical upper-layer neurons, which was due to the disruption of radial glial scaffold development. In the absence of *Fstl1*, the basal rather than the apical polarity of RGCs was altered. Our *Fstl1*^*−/−*^ mice showed no changes in the expression of the genes of the BMP, AKT/PKB, Cdc42, GSK3β, integrin or reelin signalling pathways, indicating that FSTL1 may function through a unique mechanism. Our findings will improve understanding of the molecular mechanisms of RGC development and cortical neuronal migration. Further study is required to elucidate the mechanism.

## Materials and methods

### Animals and tissue collection

Conventional disruption of *Fstl1* was achieved by crossing *Fstl1*^*fl/+*^ with *EIIa-Cre* to generate *Fstl1*^+/−^ mice, and the *Fstl1*^−/−^ mice were obtained by intercrossing the *Fstl1*^*+/−*^ mice [20, 41]. Conditional disruption of *Fstl1* in the VZ was achieved by crossing *Fstl1*^*fl/fl*^ mice with *Emx1*^*IREScre*^ mice [19] (purchased from Jackson Laboratory, Bar Harbor, Maine, USA). All animals were bred in the animal facility at Southeast University. All experiments were performed according to guidelines approved by Southeast University. Embryo gender was not considered in this study. The morning of plug detection was considered to be embryonic day 0.5 (E0.5), and the day of birth was considered to be postnatal day 0 (P0).

### Nissl and haematoxylin-eosin staining assay

Brain tissue was prepared as previously described [42], and sectioning was performed using a freezing microtome (Leica, CM 1950, 8–25 μm thick). Haematoxylin-eosin staining and Nissl staining were performed according to standard protocols.

### RNA extraction and cDNA synthesis

For E14.5 and E18.5 embryos, the dorsal cerebral cortices were dissected out, while for E12.5 embryos, the total telencephalon was removed. The tissue was immediately transferred to 500 μL of Trizol (Invitrogen) and processed for total RNA isolation according to the manufacturer’s protocol. After the RNA was purified with the RNeasy Plus Mini Kit (QIAGEN), the concentration and integrity of the RNA were analysed with the Agilent 2100 Bioanalyser (Agilent Technologies, Palo Alto, CA). The cDNA was synthesized using the M-MLV Version cDNA Synthesis Kit (Takara, Dalian, China) from 2 μg of purified total RNA.

### Quantitative real-time polymerase chain reaction

qRT-PCR was performed according to standard methods as previously described [42], and total RNA from at least three different embryos of each genotype was analysed. Specific primers for *Fstl1*, Etv5, AKT1, Cdc42, GSK3b, BMP7, Itga5, Itga6, and Itgb1 were used for qRT-PCR and are listed in Table 1.

**Table 1:** Primers for quantitative real-time PCR

	Forward	Reverse
AKT1	CGGATACCATGAACGACGTAG	GCAGGCAGCGGATGATAAAG
BMP7	GAAGTCCATCTCCGTAGTATCCG	TCTGGTCACTGCTGCTGTTTT
Cdc42	GTTGGTGATGGTGCTGTTG	CTGTGGATAACTTAGCGGTCG
Etv5	AAGAGGTTGCTCGCCGTTG	CCTTCTGCATGATGCCCTTTT
Fstl1	GCTCCCACCTTCGCCTCTAA	CCCTGCCAGCTCCACAAAA
GSK3b	TTGTCTGCCGAAATGAGTTGA	GTTTGCTTTGGGCTTGCTT
Itga5	GACCTGGGCTTAGAAACCTATT	TGAGGTTCCAGGTCTGTTTG
Itga6	TGGCCTTCTTTCTCCATCTC	CTGTCGACCCTGTGCTTTA
Itgb1	GACAGTGTGTGTGTAGGAAGAG	GCCTCCACAAATTAAGCCATTAG
Reelin	CCCAGCCCAGACAGACAGTT	CCAGGTGATGCCATTGTTGA

### In situ hybridization

The in situ hybridization was performed as previously described [12], and sections from at least three different animals of each genotype were analysed. The cDNAs were obtained as previously described. The probes for *Fstl1* and *Etv5* were amplified using the following primers: *Fstl1*: 5’-AAGGAAAAAAGCGGCCGCCCCACCTTCGCCTCTA-3’ and 5’-ACGCGTCGACATAAGATTCGCTGCCATACA-3’; and Etv5: 5’-CCGGAATTCCTGTGCTGACTCAGAAGTGCCTAAC-3’ and 5’-ACGCGTCGACAGTAAGCGAAGCCTTCGGTGTAGGG-3’.

### DiI tracing analysis

For each genotype, at least six embryonic brains were fixed in 4 % PFA for 4–8 h. Two to three microliters of DiI (1,1′-dioctadecyl 3,3,3′,3′-tetramethylindocarbocyanine perchlorate; Invitrogen; 5 % in DMSO) were injected into the lateral ventricle and allowed to diffuse at 37 °C for 7 days. Vibratome sections (150 μm) were obtained. The images include a projection of an 80-μm z-stack collected using a 20× or 40× objective on an Olympus FV1000 confocal microscope and those acquired on a standard fluorescence microscope (Olympus BX61, Tokyo, Japan).

### Immunofluorescence

Immunofluorescence experiments were performed as previously described [42]. For each genotype, at least three histological sections at three distinct rostrocaudal levels from three different animals were analysed for each immunostaining, and confocal optical sections were acquired. The primary antibodies and dilutions were as follows: rabbit anti-APC (Abcam, ab15270, 1:100), anti-BLBP (Abcam, ab32423, 1:1000), anti-CDP (M-222, Santa Cruz Biotechnology, Santa Cruz, CA, 1:500), anti-cleaved caspase-3 (Cell Signaling Technology, 9661, 1:500), anti-Ctip2 (Abcam, ab28448, 1:1000), anti-laminin A (Sigma-Aldrich, L9393, 1:2000), anti-Pax6 (Covance, 1:1000), anti-Tbr1 (Abcam, ab31940, 1:1000), and anti-Tbr2 (Abcam, ab23345, 1:800); mouse anti-beta-catenin (BD Biosciences, 1:500), anti-COUP-TFII (R&D, PP-H7147-00,2ZH7147H, 1:1000), anti-Ki67 (Leica, NCL-L-Ki67-MM1, 1:100), anti-nestin (Developmental Studies Hybridoma Bank [DSHB], Rat-401, 1:150), anti-reelin (Millipore, MAB5364, 1:3000), anti-SATB2 (Santa Cruz Biotechnology, Santa Cruz, CA, sc-81376, 1:500), and anti-ZO-1 (Invitrogen, 339100, 1:200); rat anti-BrdU (Abcam, ab6326, 1:5000), anti-histone H3 (phospho S28) (Abcam, ab10543, 1:1000), and anti-Ctip2 (Abcam, ab18645, 1:1000); and goat anti-Brn2 (C-20, Santa Cruz Biotechnology, Santa Cruz, CA, sc-6029, 1:50). The secondary antibodies included DyLight 488 donkey anti-rabbit (Thermo Fisher Scientific, SA5-10038, 1:500), Alexa Fluor 488 goat anti-rabbit (Invitrogen, A11008, 1:500), DyLight 488 donkey anti-goat (Thermo Fisher Scientific, SA5-10086, 1:500), DyLight 488 donkey anti-mouse (Thermo Fisher Scientific, SA5-10066, 1:500), Alexa Fluor 488 goat anti-rat (Invitrogen, A11006, 1:500), Alexa Fluor 555 donkey anti-rabbit (Invitrogen, A31572, 1:500), DyLight 550 donkey anti-mouse (Thermo Fisher Scientific, SA5-10067, 1:500), Alexa Fluor 546 goat anti-rat (Invitrogen, A11081, 1:500), and DyLight 650 donkey anti-rabbit (Thermo Fisher Scientific, SA5-10041, 1:500).

### BrdU analysis and birth-dating experiments

The thymidine analogue BrdU (Sigma-Aldrich, St. Louis, MO) was injected intraperitoneally into pregnant females at E12.5, E14.5 or E15.5 at a concentration of 50 mg/kg body weight. For acute BrdU labelling, embryonic brains were harvested 1 h later. For birth-dating, brains were harvested at E18.5 or P8. For BrdU immunolabelling, 8− to 25− μm sections were acid-treated with 2 M HCl for 25 min at 37 °C, carefully washed in 0.1 M PBS, and stained as described above with a monoclonal rat antibody to BrdU (Abcam, ab6326, 1:5000).

### Western blotting

The E14.5 or E12.5 dorsal cerebral cortices were collected and homogenized. The brain lysates from at least four embryos of each genotype were clarified by centrifugation at 14000 rpm. The protein concentrations were measured (Pierce Biotechnology, Rockford, IL). The brain lysates (20 μg) were subjected to 10 % SDS-PAGE and transferred to PVDF membranes. After the membranes were blocked with 5 % non-fat dry milk in Tris-buffered saline with 0.5 % Tween-20, they were incubated at 4 °C with primary antibodies, followed by incubation with the HRP-linked anti-rabbit IgG secondary antibody (Cell Signaling Technology, 7074, 1:8000) and SuperSignal West Pico Chemiluminescent Substrate (Thermo Scientific) detection. The primary antibodies were as follows: rabbit anti-phospho-Smad1 (Ser463/465)/Smad5 (Ser463/465)/Smad8 (Ser426/428) (Cell Signaling Technology, 9511, 1:4000); rabbit anti-phospho-AKT (Ser473) (Cell Signaling Technology, 4060, 1:4000); rabbit anti-AKT (Cell Signaling Technology, 9272, 1:5000); and rabbit anti-GAPDH (Cell Signaling Technology, 2118, 1:5000).

### Microscopic analysis

The images were acquired on an Olympus (Tokyo, Japan) BX61 microscope equipped with appropriate filter sets and a digital camera (DP71) or on an Olympus FV1000 confocal microscope and were processed using Image-Pro Plus 6.0.

### Morphometric analysis and statistics

For morphometric analysis, at least three embryos were analysed for each condition in parallel experiments. Both hemispheres of at least three matching sections from each brain were used for the measurements, and comparisons between the mutants and the control littermates were made using more than three litters. The cells were counted in a radial segment that was 200 or 300 μm in width and spanned from the ventricular surface to the pia. For the proliferation assay, the number of BrdU^+^ cells in each radial unit was counted. For the birth-dating experiments, a single optical section of a confocal microscope image was used, and the number of BrdU^+^ cells in ten equally divided bins that spanned the cortical thickness was counted and expressed as a fraction of the total number of BrdU^+^ cells per bin. To evaluate the distribution of Cux1^+^ neurons, sections were divided into ten bins of equal height and 300 μm in width. Bins 1–3, 4–6 and 7–9 were grouped as upper, middle and lower, respectively. All of the data were statistically analysed with Microsoft Office Excel and graphed using GraphPad Prism software. The error bars represent the standard error of the mean. Two-tailed Student’s *t*-test was used to analyse statistical significance (* *P* ≤ 0.05, ** *P* ≤ 0.01, *** *P* ≤ 0.001).

## Additional files

Additional file 1: Figure S1. The neurogenesis of upper-layer neurons was not affected by *Fstl1* deletion. (A to D’) IPCs at E13.5 (A to B’), E14.5 (C to C’) and E15.5 (D to D’) were labelled by immunostaining with an anti-Tbr2 antibody. The number and distribution of the Tbr2^+^ IPCs were similar between the *Fstl1* ^*−/−*^ (A’, B’, C’ and D’) and the WT (A, B, C and D) cortices. Scale bars: 100 μm. (PDF 420 kb) Additional file 2: Figure S2. The integrity of the pial BM was not affected by the deletion of *Fstl1*. (A to A’) A robust, continuous band of laminin A immunoreactivity was detected in both the *Fstl1* ^*−/−*^ (A’) and WT (A) cortices. Scale bars: 50 μm. (PDF 53 kb) Additional file 3: Figure S3. Analysis of the possible mechanisms by which *Fstl1* regulates the development of radial glial processes. (A) Western blotting for phospho-Smad1/5/8, AKT and phospho-AKT (S473). Similar phosphorylation levels of Smad1/5/8 and AKT in the *Fstl1* ^*−/−*^ and WT brains at E14.5. The expression of AKT in the *Fstl1* ^*−/−*^ cortices did not differ from that in the WT cortices. (B to C) Quantification of the mRNA levels of BMP7 (B) and AKT1 (C) at E14.5 showed no differences. (D and D’) Etv5 mRNA was predominantly expressed at E14.5 in the VZ of the *Fstl1* ^*−/−*^ mice (D’), similar to the expression in the WT mice (D). (E and F) The transcription levels of Etv5 (D) and reelin (F) mRNA did not differ between the WT and *Fstl1* ^*−/−*^ mice. (F) (G and G’) The distribution of reelin^+^ CR cells in the *Fstl1* ^*−/−*^ mice also did not differ from that in the WT mice. (H to L) Quantification of the mRNA levels of Cdc42 (H), GSK3β (I) and integrinβ1/α5/α6 (J to L) by RT-PCR showed similar mRNA levels in the WT and *Fstl1* ^*−/−*^ mice. Scale bars: 50 μm for D and D’; 100 μm for F and F’. (PDF 723 kb)

## Abbreviations

APC: Adenomatosis polyposis coli
BLBP: Brain lipid-binding protein
BM: Basement membrane
BMP: Bone morphogenetic protein
BrdU: 5-bromo-2-deoxyuridine
Brn2: POU domain transcription factor 2
cdc42: Cell division cycle 42
CGE: Caudal ganglionic eminence
COUP-TFII: Ovalbumin upstream promoter transcription factor II
CR: Cajal-Retzius
Ctip2: Chicken ovalbumin upstream promoter transcription factor-interacting protein 2
Cux1: Cut-like homeobox 1
CXCL12: Chemokine (C-X-C motif) ligand 12
Etv5: Ets variant 5
FGF: Fibroblast growth factor
Foxp2: Forkhead box protein 2
Fstl1: Follistatin like-1
GAPDH: Glyceraldehyde-3-phosphate dehydrogenase
GSK3β: Glycogen synthase kinase 3 beta
HRP: Horseradish peroxidase
IPCs: Intermediate progenitor cells
Itga: Integrin alpha
Itgb1: Integrin beta-1
IZ: Intermediate zone
Lhx6: LIM homeobox protein 6
MAPK: Mitogen-activated protein kinase
MGE: Medial ganglionic eminence
PAGE: Polyacrylamide gel electrophoresis
Pax6: Paired box gene 6
pHH3: Phospho-histone H3
PKB: Protein kinase B
PVDF: Polyvinylidene difluoride
RGCs: Radial glial cells
SDS: Sodium dodecyl sulphate
SST: Somatostatin
Tbr1: T-box brain gene 1
Tbr2: T-box brain gene 2
VZ: Ventricular zone
ZO-1: Tight junction protein 1
