# Supplementary figures and images for: Fstl1 is involved in the regulation of radial glial scaffold development
Source: Mol Brain. 2015 Sep 17;8:53. doi: 10.1186/s13041-015-0144-8 (PMC4573935; doi:10.1186/s13041-015-0144-8)

**Fstl1<sup>+/+</sup>**

**Fstl1<sup>-/-</sup>**

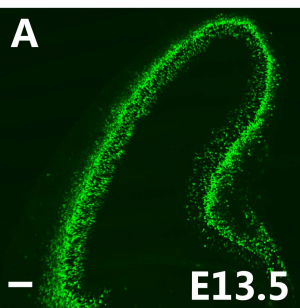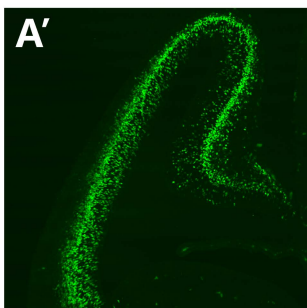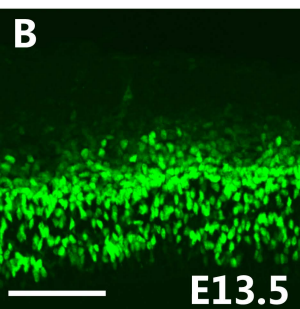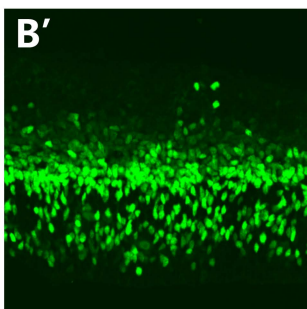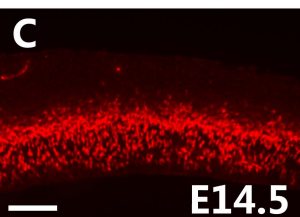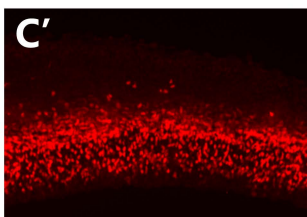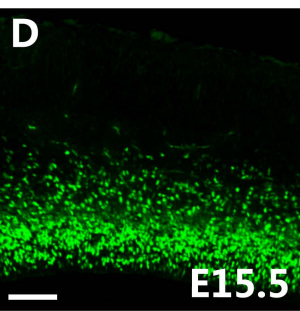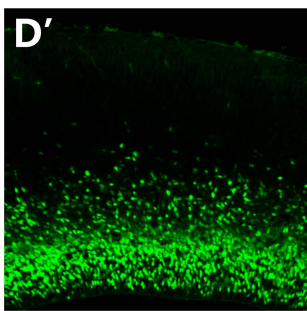

Supplement: Additional file 1: Figure S1. — The neurogenesis of upper-layer neurons was not affected by Fstl1 deletion. (A to D’) IPCs at E13.5 (A to B’), E14.5 (C to C’) and E15.5 (D to D’) were labelled by immunostaining with an anti-Tbr2 antibody. The number and distribution of the Tbr2+ IPCs were similar between the Fstl1 −/− (A’, B’, C’ and D’) and the WT (A, B, C and D) cortices. Scale bars: 100 μm. (PDF 420 kb) [file 13041_2015_144_MOESM1_ESM.pdf]

**Fstl1<sup>+/+</sup>**

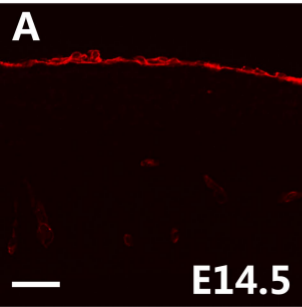

**Fstl1<sup>-/-</sup>**

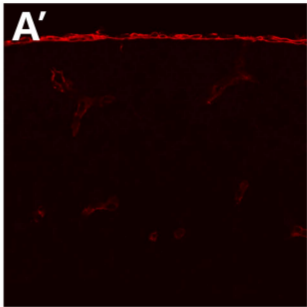

Supplement: Additional file 2: Figure S2. — The integrity of the pial BM was not affected by the deletion of Fstl1. (A to A’) A robust, continuous band of laminin A immunoreactivity was detected in both the Fstl1 −/− (A’) and WT (A) cortices. Scale bars: 50 μm. (PDF 53 kb) [file 13041_2015_144_MOESM2_ESM.pdf]

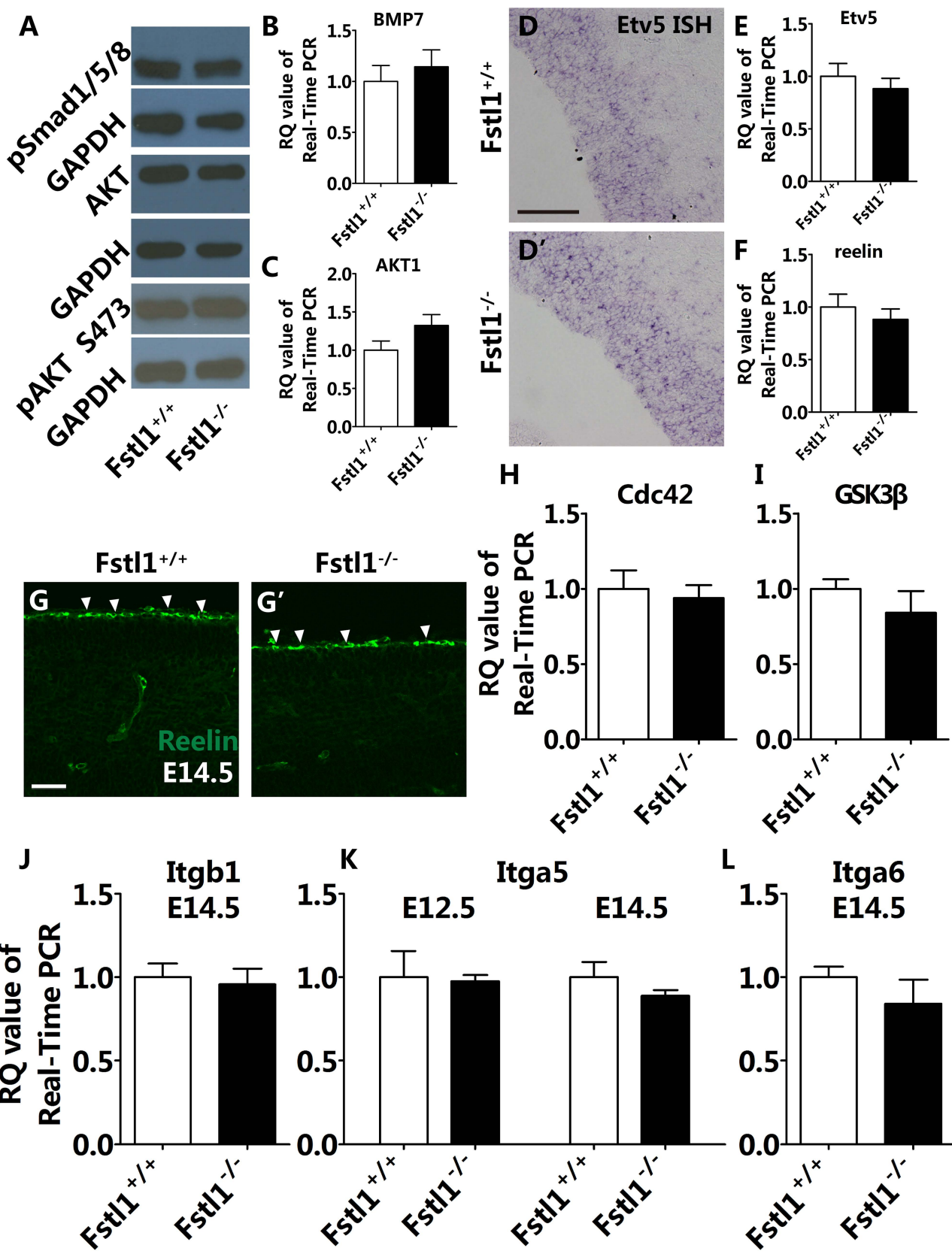

Supplement: Additional file 3: Figure S3. — Analysis of the possible mechanisms by which Fstl1 regulates the development of radial glial processes. (A) Western blotting for phospho-Smad1/5/8, AKT and phospho-AKT (S473). Similar phosphorylation levels of Smad1/5/8 and AKT in the Fstl1 −/− and WT brains at E14.5. The expression of AKT in the Fstl1 −/− cortices did not differ from that in the WT cortices. (B to C) Quantification of the mRNA levels of BMP7 (B) and AKT1 (C) at E14.5 showed no differences. (D and D’) Etv5 mRNA was predominantly expressed at E14.5 in the VZ of the Fstl1 −/− mice (D’), similar to the expression in the WT mice (D). (E and F) The transcription levels of Etv5 (D) and reelin (F) mRNA did not differ between the WT and Fstl1 −/− mice. (F) (G and G’) The distribution of reelin+ CR cells in the Fstl1 −/− mice also did not differ from that in the WT mice. (H to L) Quantification of the mRNA levels of Cdc42 (H), GSK3β (I) and integrinβ1/α5/α6 (J to L) by RT-PCR showed similar mRNA levels in the WT and Fstl1 −/− mice. Scale bars: 50 μm for D and D’; 100 μm for F and F’. (PDF 723 kb) [file 13041_2015_144_MOESM3_ESM.pdf]
